# Impaired DNA Repair Fidelity in a Breast Cancer Patient With Adverse Reactions to Radiotherapy

**DOI:** 10.3389/fpubh.2021.647563

**Published:** 2021-06-07

**Authors:** Ghazi Alsbeih, Najla Al-Harbi, Sheikh Ismail, Michael Story

**Affiliations:** ^1^Radiation Biology Section, Biomedical Physics Department, King Faisal Specialist Hospital and Research Centre, Riyadh, Saudi Arabia; ^2^Experimental Radiation Oncology Department, University of Texas M.D. Anderson Cancer Center, Houston, TX, United States; ^3^College of Medicine, Alfaisal University, Riyadh, Saudi Arabia; ^4^Commercialization & Entrepreneurship Department, Texas A&M University, Bellaire, TX, United States; ^5^Radiation Oncology Department, University of Texas Southwestern Medical Centre, Dallas, TX, United States

**Keywords:** DNA double-strand breaks, misrepair, *Not*I fragment, *Alu* sequence, radiosensitivity, adverse reactions to radiotherapy, repair fidelity, pulsed field gel electrophoresis

## Abstract

We tested the hypothesis that differences in DNA double-strand break (DSB) repair fidelity underlies differences in individual radiosensitivity and, consequently, normal tissue reactions to radiotherapy. Fibroblast cultures derived from a radio-sensitive (RS) breast cancer patient with grade 3 adverse reactions to radiotherapy were compared with normal control (NC) and hyper-radiosensitive ataxia-telangiectasia mutated (ATM) cells. DSB repair and repair fidelity were studied by Southern blotting and hybridization to *Alu* repetitive sequence and to a specific 3.2-Mbp *Not*I restriction fragment on chromosome 21, respectively. Results for DNA repair kinetics using the *Not*I fidelity assay showed significant differences (*P* < 0.001) with higher levels of misrepaired (misrejoined and unrejoined) DSBs in RS and ATM compared with NC. At 24-h postradiation, the relative fractions of misrepaired DSBs were 10.64, 23.08, and 44.70% for NC, RS, and ATM, respectively. The *Alu* assay showed significant (*P* < 0.05) differences in unrepaired DSBs only between the ATM and both NC and RS at the time points of 12 and 24 h. At 24 h, the relative percentages of DSBs unrepaired were 1.33, 3.43, and 12.13% for NC, RS, and ATM, respectively. The comparison between the two assays indicated an average of 5-fold higher fractions of misrepaired (*Not*I assay) than unrepaired (*Alu* assay) DSBs. In conclusion, this patient with increased radiotoxicity displayed more prominent misrepaired than unrepaired DSBs, suggesting that DNA repair fidelity is a potential marker for the adverse reactions to radiotherapy. More studies are required to confirm these results and further develop DSB repair fidelity as a hallmark biomarker for interindividual differences in radiosensitivity.

## Introduction

Compelling evidence suggests that adverse reactions to radiotherapy are associated with increased patient sensitivity to ionizing radiation (1). Likewise, individual variation in radiosensitivity is well-recognized and at least partly determined by genetic factors, as clinical, epidemiologic, and laboratory data have indicated (2–6). Initial evidence for the heritability of radiosensitivity originated from the studies of rare genetic disorders such as ataxia-telangiectasia (A-T), Nijmegen breakage syndrome, Nijmegen breakage syndrome-like disorder (RAD50 deficiency), ligase IV deficiency, A-T-like disorder, and Fanconi's anemia (7, 8). Although each syndrome has its own phenotypical characteristics, cells derived from those patients demonstrate spontaneous chromosomal instability and hypersensitivity to ionizing radiation due to mutations affecting DNA strand breaks signaling, recognition, and repair capability (9).

Between the multiple damages produced by ionizing radiation, DNA double-strand breaks (DSBs) are the main critical lesions and are highly consequential for genome integrity (10). Repair is a fundamental inherent mechanism of genome protection, and the ability to rejoin DSBs with appropriate fidelity determines cell fate, recovery, death, or mutagenesis (11). Unrejoined and misrejoined DSBs are important lesions for radiation-induced cell killing, although the relationship between DNA repair, misrepair, and cell survival is not fully understood (12). Misrepaired (unrejoined or misrejoined) DSBs can lead to chromosome aberrations and micronucleus formation, and both endpoints generally correlate with the degree of cell killing (13). However, cellular death mechanisms, cell cycle kinetics, and the various underlying genetic defects influence the expression and detection of chromosome damage, thus making qualitative cytogenetic approaches less precise as quantitative measures of cellular radiosensitivity (14). Another method for examining the fidelity of DNA repair is to measure the ability of cells or cell extracts to reactivate plasmids containing damaged reporter genes. This approach has proven useful for examining DNA repair in some radiosensitive cell lines (15); however, it is also not particularly quantitative, as there was little difference in this measure of DSB repair fidelity between some cell lines with wide differences in radiosensitivity (16).

Another appealing DNA repair fidelity technique that assesses DSB misrepair has been described (17, 18). The procedure relies on the use of endonucleases to cleave out specific DNA fragments that are subjected to pulsed-field gel electrophoresis and detected by Southern hybridization to a known probe. Although it is not a widely used technique, the few data obtained using this method were scientifically interesting, as it allowed the detection of differences in the proportion of correctly rejoined DSBs produced by irradiations of different LETs (19) and in the fraction of unrejoined DSBs in cell lines of different origins (20). We have previously used this technique to detect misrepair of radiation-induced DSBs in a patient with unidentified chromosomal fragility syndrome and a family history of radiosensitivity (21). Here, we extend this basic research work to examine the ability of this technique to detect differences in DSB repair fidelity in a fibroblast cell line derived from a breast cancer patient who developed marked late adverse reactions to radiotherapy. The results were compared with a cell line of a patient with no radiotherapy tissue reactions and an extremely radiosensitive A-T mutated (ATM) cell line.

## Materials and Methods

### Cell Cultures, Patients, and Ethical Considerations

Three primary non-transformed human skin fibroblast cultures, normal control (NC), radiosensitive (RS), and ATM were used. The ethics committee of the institutional review board approved the study (CA-06294/16672/50192; 1990). The ATM cell line (GM01588A) was purchased from the American Type Culture Collection (Manassas, VA, USA). The NC and RS were derived from two breast cancer patients, as described previously (22). Briefly, the NC patient did not develop any discernable (grade 0; Radiation Therapy Oncology Group and the European Organization for Research and Treatment of Cancer grading system) adverse effects, whereas the RS patient developed marked (grade 3) skin atrophy and telangiectasia. Both patients were treated by definitive radiotherapy (50 Gy in 2 Gy fractions). Compared with NC, RS was considered to have excessive late reactions for the dose received, which was verified from their treatment and dosimetry records. The cumulative total dose delivered to different normal tissues was estimated from computed tomography treatment plans. Late effects were documented from patient records. The median follow-up was 19 months (range: 13 to 25) at the time of data collection. The *in vitro* radiosensitivity characterization of the cell strains using clonogenic survival curves was published previously (23, 24). Briefly, the surviving fraction at 2 Gy radiation dose was 0.34 [95% confidence interval (CI) = 0.31–0.37], 0.18 (95% CI = 0.13–0.25), and 0.03 (95% CI = 0.02–0.04) for NC, RS, and ATM cell strains, respectively. The three cell lines were maintained in alpha minimal essential medium supplemented with 15% fetal bovine serum. Experiments were performed with contact-inhibited cultures to minimize cell cycle-dependent variations in DNA repair. All incubations were performed at 37°C in a humidified atmosphere of 5% carbon dioxide.

### Irradiation

Cells were irradiated on ice in 150-mm Petri dishes using a ^137^Cs source at a dose rate of 3.65 Gy/min.

### *Not*I and *Alu* DNA Double-Strand Breaks Repair Assays

The *Not*I repair fidelity assay involves the use of endonuclease to cleave out a specific DNA fragment that can be detected by Southern hybridization to a known probe. Using the *Not*I rare cutting restriction enzyme, a unique 3.2-Mbp restriction fragment is cleaved out of the long arm of chromosome 21. After subjecting DNA to pulsed-field gel electrophoresis, the *Not*I 3.2-Mbp fragment migrates as a single band, which is detected and quantified by hybridization to the D21S1 single copy probe. Unrejoined and misrejoined *Not*I fragments induced by irradiation migrate separately from the *Not*I band. The extent of incomplete restoration of the 3.2-Mbp *Not*I band is taken as a quantitative measure of misrepair in this specific region of the genome. The *Alu* assay follows the same principal except for the endonuclease use; being a highly repetitive sequence, it assesses unrepaired DSBs in the whole genome.

The Southern blot procedures for *Not*I and *Alu* DNA repair assays were published in detail previously (21, 25). Briefly, confluent cells were irradiated with either 30 Gy (for *Alu* genomic probe) or 80 Gy (for *Not*I specific fragment probe) and incubated at 37°C for up to 24 h. The choice of radiation doses was determined in preliminary experiments and optimized to induce DSBs in approximately 80% of the target DNA in each of the *Alu* and *Not*I fragments (21). The cells were trypsinized and centrifuged. The pellet was resuspended at a concentration of 2–4 × 10^7^ cells/mL, for *Not*I DSBs repair fidelity, or 10^5^ cells/mL for *Alu* total genomic DSB repair assays. The cell suspension was mixed with 1% low-melting point agarose (InCert, FMC BioProducts) and poured into plastic molds. Solidified plugs were lysed [0.5-M ethylenediaminetetracetic acid disodium (Na_2_EDTA), 1% sodium lauroyl sarcosine, 1 mg/mL proteinase K, pH 8] at 50°C for 2–3 days, washed and stored in 0.5-M Na_2_EDTA (pH 8) at 4°C. For restriction enzyme digestion, DNA in a half-plug was digested with 25 units of *Not*I restriction enzyme (Promega, Madison, WI, USA) at 37°C overnight and inactivated at 50°C for 2 h. As for genomic DNA repair (*Alu*), there is no need for a restriction enzyme treatment.

DNA DSBs were separated by pulsed-field gel electrophoresis, using a CHEF Mapper or CHEF-DR II electrophoresis system (Bio-Rad), in a 0.5× Tris/borate/EDTA buffer (45-mM Tris-base, 45-mM boric acid, 1-mM Na_2_EDTA; pH 8). The field strength was 1.5 V/cm with pulse times linearly increasing from 50 to 5,000 s. Electrophoresis was carried out for 18 h at 24°C for total genomic DNA (*Alu* assay) and at 12°C for 140 h for DNA repair fidelity (*Not*I assay). Gels were stained with ethidium bromide for 15 min, destained for 1 h, and photographed with a digital camera system under ultraviolet transillumination. *Schizosaccharomyces pombe* and *Hansenula wingei* chromosomes served as size markers. For DNA transfer, gels were exposed to a germicidal ultraviolet lamp and soaked in the alkaline transfer solution (0.4-M NaOH; 0.6-M NaCl) for 30 min, and the DNA was transferred by capillary action to nylon membranes (GeneScreen Plus, Du Pont, NEN Research Products, Boston, MA, USA) over 2 days and air-dried.

For hybridization to the 3.2-Mbp *Not*I restriction fragment, the plasmid containing the D21S1 probe (pPW228C) was isolated from the host bacteria; the insert was cut out with *Eco*RI and gel-purified by standard procedures. Radioactively labeled probe was produced *via* random priming kit (Boehringer Mannheim, Gaithersburg, MD, USA) using [α-^32^P]dCTP (222 TBq/mmol, NEN Life Science Products, Boston, USA). The membranes were hybridized for 2–2.5 days at 45°C in Hybrisol I (Intergen, Burlington, MA, USA) and heat-denatured ^32^P-labeled DNA probe (0.3–1 × 10^8^ cpm/membrane). The membranes were washed and exposed to storage phosphor screens for 1 to 6 days, depending on the signal intensity of each membrane. Likewise, the *Alu* probe was prepared and processed in a similar way except that it was cut out of hosting plasmid (BLUR8) using *Bamh1* restriction enzyme, and the labeled probe was hybridized overnight. At least two experiments were carried out for each cell line, two samples from each experiment were run on separate gels, and the results were pooled.

### Data Analysis

The quantitative analysis of the Southern blot data was described previously (21). Briefly, the total intensity (*I*_0_) was calculated as the integral of the signal in the whole lane after baseline adjustment. For DNA repair in the whole genome (*Alu* assay), the intensity of the migrating DSBs in the *Alu* sequence *(I*_1_) was quantified by the integral of the signal below the well. The relative amount of DNA released from the plugs (corresponding to DSBs ≤ 10–12 Mbp) was calculated by dividing the signal intensity of the migrating DNA *(I*_1_) by that of the whole lane, including the wells *(I*_0_). For DSB repair fidelity (*Not*I assay), the intensity of the full-length *Not*I fragments (*I*) was quantified by the integral of the signal in the 3.2-Mbp band. The signal intensity of misrepaired (misrejoined and unrejoined) DSBs (*I*_1_) was calculated by subtracting *I* from *I*_0_. The relative amount of misrepaired 3.2-Mbp *Not*I restriction fragment in each lane was calculated by dividing *I*_1_ by *I*_0_. For both assays, the kinetics of DSB repair, after various repair times, was presented as a fraction (%) of DSBs remaining unrepaired or misrepaired (also known as the fraction of “radiation” activity released). The DSBs remaining unrepaired (Alu assay) or misrepaired (NotI assay) were calculated by dividing the relative amount by that induced before any repair (0 h repair time) after subtraction of background (0 Gy) for each. Testing for statistically significant differences in the kinetics of DSBs misrepaired or remaining unrepaired between the cell strains was carried out using the one-way repeated measures analysis of variance test. This test compares differences in the mean values, computed over all the repair time points, among the cell strains. Comparing between the cell strains at each time point of repair was carried out using the one-way analysis of variance test. Testing for the difference from baseline zero-level was conducted using a one-tailed *t*-test. SigmaPlot software (versions 12.5 and 13; Systat Software Inc., San Jose, CA, USA) was used for statistical analysis. *P* < 0.05 was considered significant.

## Results

**Figure 1:**
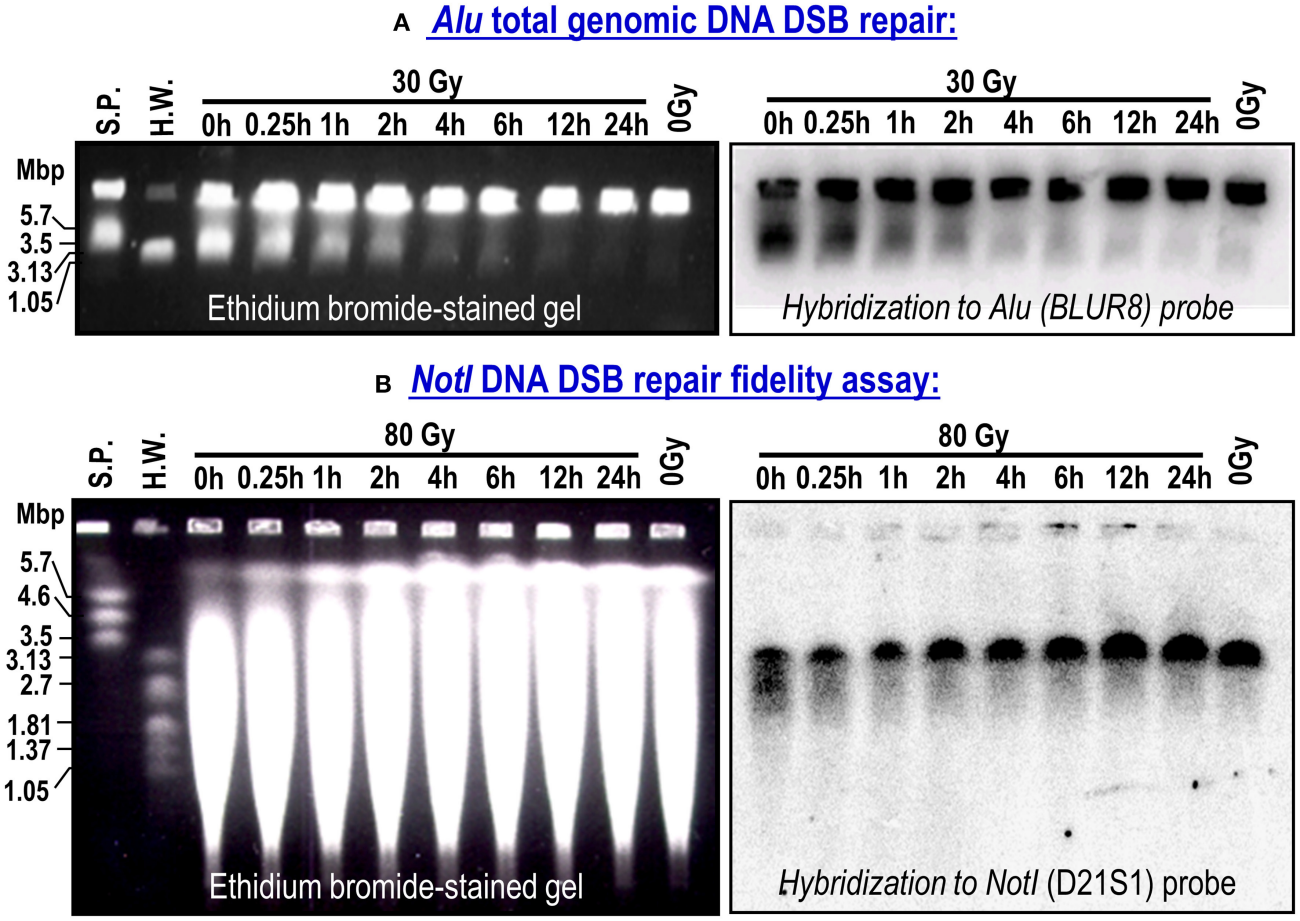
Representative ethidium bromide-stained gels and corresponding membranes hybridized to *Alu* repetitive sequence in whole genome (BLURB probe; **A**) or specific *Not*I fragments located on chromosome 21 (D21S1 probe; **B**). Bulk rejoining of DSBs in genomic DNA and DSB repair fidelity in a 3.2-Mbp DNA fragment. Data from NC cell strain. S.P. (*S. pombe*) and H.W. (*H. wingei*) are DNA size standards.

Figure 1 shows representative examples of ethidium bromide-stained gels (left panels) and the corresponding membranes hybridized to the *Alu* (BLUR8) and *Not*I (D21S1) probes (right panels). On the gel photographs, we can distinguish the wells containing non-migrating high molecular weight DNA and a smear of migrating DSBs of approximately 5.7 Mbp or less. On the membranes, we only see the DNA hybridized with the probes. The *Alu* genomic probe hybridizes to the widespread *Alu* sequence in the human genome. The signal below the wells (plugs) represents the amount of DSBs in the whole genome. The D21S1 probe hybridizes to a specific 3.2-Mbp *Not*I fragment on chromosome 21. Full-length *Not*I fragments are located in a band at 3.2 Mbp. Irradiation breaks down the *Not*I fragments leading to a smear seen below the *Not*I band. When the time for repair increases from 0 to 24 h, the broken DSBs are repaired, resulting in the gradual diminishing of the *Alu* signal below the wells and restitution of the full-length *Not*I fragments.

**Figure 2:**
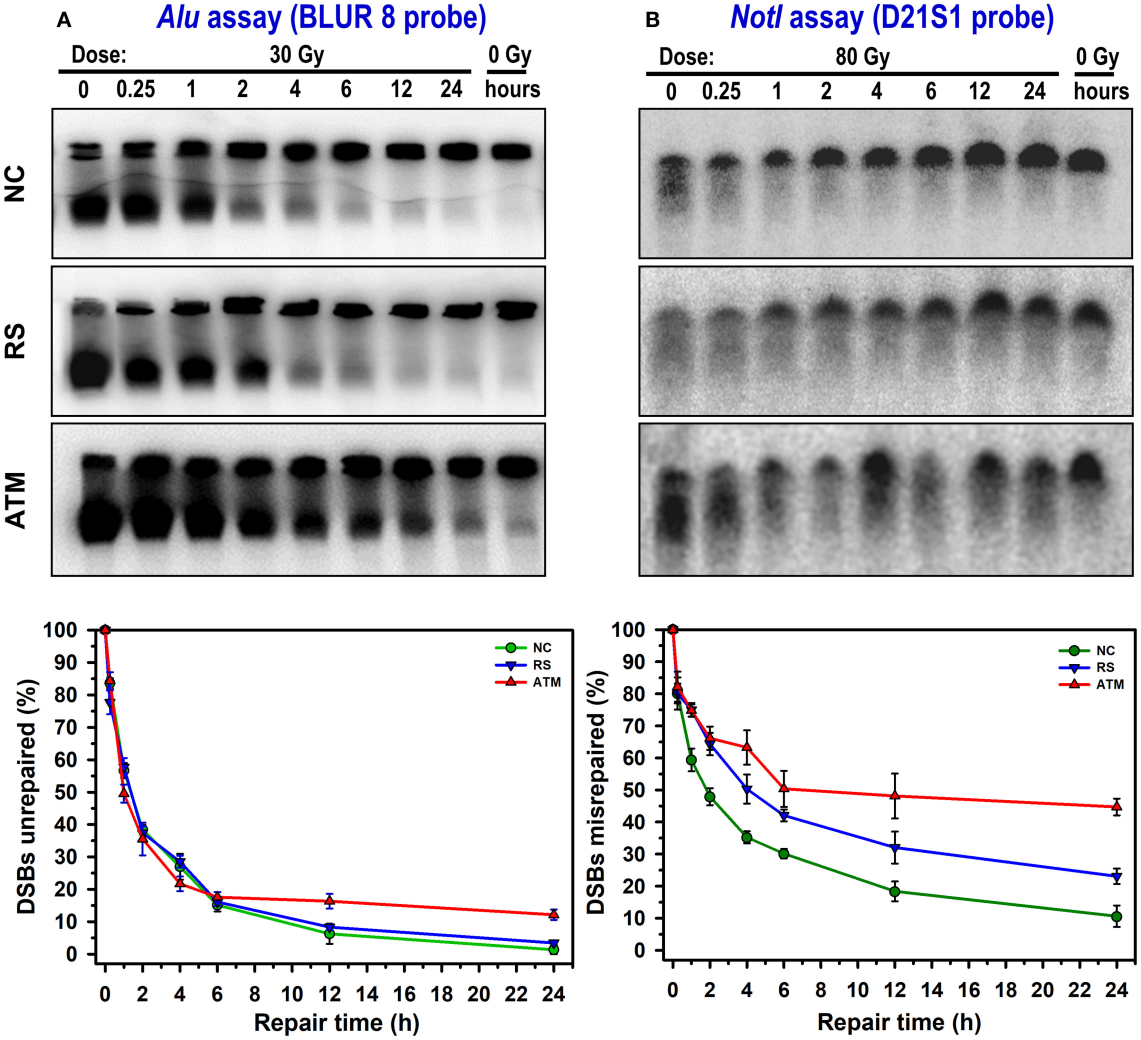
Representative examples of DSB repair and repair fidelity in NC, RS, and ATM cell strains. Membranes were hybridized to *Alu* repetitive sequence (whole-genome DSBs unrepaired; **A**) and specific *Not*I fragments (DSBs misrepaired; **B**). Curves show corresponding kinetics of DSB repair after subtraction of membrane background and normalizing to total amount of DSBs induced immediately after irradiation (0-h repair time). Data points represent mean, and error bars represent standard error of mean. Results of statistical analysis (one-way repeated measures analysis of variance) are as follows: *Not*I assay, there is an overall significant difference between cell lines (*P* < 0.001). Pairwise comparison: ATM *vs*. NC: *P* < 0.001; RS *vs*. NC: *P* = 0.014; ATM *vs*. RS: *P* = 0.034. For *Alu* assay, no overall significant differences between cell lines (*P* = 0.82). However, significant differences (one-way analysis of variance) between cell lines (*P* < 0.05) were observed at 12 and 24 h. Pairwise comparison at 12 h: ATM *vs*. NC: *P* = 0.024; ATM *vs*. RS: *P* = 0.054; RS *vs*. NC: *P* = 0.548. Pairwise comparison at 24 h: ATM *vs*. NC: *P* < 0.001; ATM *vs*. RS: *P* < 0.001; RS *vs*. NC: *P* = 0.287.

Illustrative comparison of *Alu* and *Not*I hybridized membranes in the three cell lines are shown in the upper panels of Figure 2. With increasing time for repair, the hybridization signals were different between the cell lines. The control cell line showed the highest restitution of DSBs compared with the radiosensitive patient and particularly the extremely sensitive ATM cell strain that showed a comparatively high level of unrepaired DSBs. Although the average background level of DSBs (0 Gy) was 7.31% [range: 6.48–9.36%; standard deviation (SD) = 1.79] in the *Alu* assay, it was 27.47% (range: 21.76–35.28%; SD = 6.99) in the *Not*I assay. In the *Alu* whole-genome assay, the fraction of induced DSBs, without repair, after a dose of 30 Gy was 0.84 (SD = 0.02), 0.87 (SD = 0.02), and 0.83 (SD = 0.03) for NC, RS, and ATM, respectively. Similarly, in the *Not*I fragments, a dose of 80 Gy induced comparable fractions in the cell strains [NC: 0.79 (SD = 0.04), RS: 0.83 (SD = 0.05), and ATM: 0.84 (SD = 0.03)].

The results for DSBs remaining unrepaired or misrepaired after subtraction of 0 Gy and normalizing to the induction level (0 h) are presented in the lower panels of Figure 2. The repair kinetics indicated differences not only between the three cell strains but also between genomic *Alu* and specific *Not*I repair assays. Although it is obvious that these cell lines display a wide range of radiosensitivity exhibited by different capacities to restitute broken DNA, the *Not*I repair fidelity assay showed higher levels of misrepaired DSBs. For example, at 24 h of repair, where the largest differences are seen, the average values for residual DSBs were 5.60% (SD = 5.73) for *Alu* and 26.13% (SD = 17.25) for *Not*I assays. This is an average of a 5-fold increase in residual DSBs detection in the DNA repair fidelity assay compared with general or bulk DNA repair. In addition, using a one-tailed *t*-test to examine the divergence of the 24-h residual DSBs in the three cell strains from the baseline (0%) level, the differences for the *Not*I assay were statistically significant (*P* = 0.03), although not so for the *Alu* assay (*P* = 0.08).

The percentage of DSBs misrepaired or remaining unrepaired in the *Not*I fragments after 24 h of repair were 10.64, 23.08, and 44.70% for NC, RS, and ATM, respectively. This indicates a 2.2- and 4.2-fold increase in misrepair in RS and ATM as compared with NC, respectively. Statistical analysis of *Not*I DNA repair kinetics showed a significant difference in DSBs misrepaired between the three cell strains (one-way repeated measures analysis of variance, *P* < 0.001). In comparison, the *Alu* assay showed lower levels of DSBs remaining unrepaired. The NC and RS showed similar repair kinetics, whereas ATM displayed a relatively higher level of unrepaired DSBs. The percentages of the DSBs unrepaired at 24 h in the *Alu* whole-genome assay were 1.33, 3.43, and 12.13% for NC, RS, and ATM, respectively. This indicates a 2.6- and 9.3-fold increase in DSBs unrepaired in RS and ATM as compared with NC, respectively. Nevertheless, there was no statistically significant difference in unrepaired DSBs between the three cell strains when all time points are considered together (one-way repeated measures analysis of variance, *P* = 0.82). However, testing for dissimilarities at each time point of repair kinetics revealed statistically significant differences at 12 and 24 h between ATM and both NC and RS (one-way analysis of variance, *P* < 0.05); meanwhile, no difference was observed between NC and RS (Figure 2).

## Discussion

In this study, we examined the possibility of detecting differences in DSB misrejoining (unrepaired and misrepaired) in a primary human fibroblast strain derived from a clinically radiosensitive patient with marked (grade 3) adverse effects to radiotherapy. The results were compared with a cell line from a patient who exhibited no adverse effects (grade 0) and a cell line derived from an ATM patient who is known for their extreme radiosensitivity (patients typically develop grade 4 adverse reactions). In the three cell lines, a dose of 30 Gy led to the induction (before any repair) of comparable amounts of DSBs in the entire genome (*Alu* probe, 0.85, SD = 0.02). Similarly, a dose of 80 Gy produced comparable amounts of DSBs in the *Not*I fragments (0.81, SD = 0.02). These results are in line with results obtained by other investigators (26). However, when irradiated cells were allowed to repair, these different cell lines displayed a distinguishable capacity to join DSB ends (Figure 2). The repair kinetics displayed an exponential decay shape with an initial rapid decrease in the fraction of DSBs misrepaired or unrepaired that extends to approximately 4 h, followed by a slight decrease after that. This shape is quite common for DSBs repair kinetics, which can be mathematically fitted by first-order or biphasic exponential decay equations (27, 28). Although such a fitting may facilitate analysis and contribute to the characterization of hypothetical biological processes, it is not required to derive conclusions from the results presented (29).

The three cell strains showed comparable initial repair kinetics for the entire genome (*Alu* assay), whereas differences were mainly detectable in the slow distal time points from 12 to 24 h (Figure 2). The fraction of DSBs remaining unrepaired was between 1 and 12% of the initial damage (i.e., 88 to 99% were rejoined). However, this level does not seem to significantly diverge from the baseline (0%) level (*P* = 0.08). Note that the NC and the RS cell lines showed similar kinetics when compared with ATM, which seemed to have a slightly faster repair up to 4 h, followed by slower repair after that. Although there were no overall significant differences between the three cell strains (*P* = 0.82), the ATM showed, as expected, significantly (*P* < 0.05) higher unrepaired DSBs at 12 and 24 h postradiation. In contrast, for the repair fidelity assay, the three cell strains showed distinguishable kinetic curves of statistical significance (*P* < 0.001) at much earlier repair times, which continued to 24 h. On average, the *Not*I assay showed a 5-fold higher level of residual DSBs than the *Alu* assay. Therefore, the repair fidelity assay was able to detect differences in DSB repair between patients with and without radiotoxicities and the extreme radiosensitivity of ATM. This is consistent with a previous observation of normal, ATM, and AT-heterozygous cell types (26). The NC cell line exhibited the lowest rate of misrepair (highest repair fidelity) in the 3.2-Mbp *Not*I restriction fragment with a level of misrejoining and unrejoining at 24 h of 11% of the initial damage, i.e., 89% of ends were rejoined. The ATM cell line showed a relatively high level of misrepair, 45% (55% of rejoining), whereas the RS cell line was intermediate with 23% (77% rejoining) misrepair. The latter is a relatively lower misrepair in comparison with a previously reported radiosensitive cell strain, which showed a *Not*I DSBs misrepair level at 24 h of 37% (21). Furthermore, these levels of DSB misrepair at 24 h seem to significantly diverge from the baseline (0%) level (*P* = 0.03).

The results suggest that, for the radiosensitive patient, misrepair of broken DNA ends in non-transformed fibroblasts is associated with late normal tissue reactions after radiotherapy. In view of the results with a normally sensitive patient, a radiosensitive patient who developed late adverse complications, and an ATM patient (GM01588), who will invariably develop severe and even fatal complications if treated with a standard radiotherapy regimen, it is tempting to speculate that the misrepair assay could provide a better resolution and more accurate measure of radiosensitivity than DNA repair within the entire genome. Nevertheless, a previous work using the *Not*I assay identified differences in misrepair only after fractionated irradiation (26). Using normal (derived from a healthy volunteer with no radiosensitivity data), ATM, and AT heterozygous cell lines, differences were observed after low dose per fraction (5 and 10 Gy) and not with a high (20 Gy) or at the total 80 Gy given as a single dose. In our study, the variation was observed after a single 80-Gy dose. Other doses were not examined. The apparent discrepancy may lay in the levels of misrejoined DSB observed in the normal cell strains. However, the dissimilarities in the methodological details between the two studies preclude a precise comparison.

Although this study is not designed to delve into the particular radiosensitivity of the ATM cell strain, it can be speculated from the data that radiosensitivity is associated with an increased level of misrepaired DSBs. This is in line with the observation that it displayed increased levels of unrepaired DSBs (30). The present demonstration of a higher level of misrejoining observed in the *Not*I repair fidelity assay (Figure 2) supports a mechanistic answer for the increased rate of chromosomal aberrations in ATM and other chromosomal fragility syndromes (24). Genome editing technology demonstrated that artificially introduced heterozygous mutations of the ATM gene increased the number of chromosomal aberrations after irradiation and shed light on the genetic basis underlying individual differences in radiosensitivity within the human population (31). In addition, researchers described ATM cells as having higher initial repair speed followed by a stagnant slow repair leading to higher DSBs unrepaired in the whole genome (30), which can be perceived in this study (Figure 2, left panels). However, this is not the case in the repair fidelity assay, which suggests slower initial repair kinetics than that observed with NC and RS cell strains (Figure 2, right panels).

Finally, this study contributes to the ever-expanding experimental evidence for the increased radiosensitivity of a small percentage of radiotherapy patients treated with standard regimens. Those patients develop adverse radiation sequelae that cannot be attributed to dose distribution or volume irradiation (22). Many reports about cohorts and individual cases of radiosensitivity have been published where clinical radiosensitivity was associated with certain *in vitro* experimental endpoints with variable results (14). Between the various mechanistic pathways investigated, the radiation-induced DNA damage response remains the most well-characterized (32, 33). However, some other mechanisms and pathways were suggested to be involved in patient radiosensitivity, including oxidative stress, stem cell response, activation of inflammation pathways with the secretion of cytokines, genetics, non-coding RNA, and potentially epigenetic factors that can be studied using a large number of functional assays (3). For instance, individual variations in radiosensitivity have been attributed to a dissimilar genetic makeup affecting various cellular processes (2). However, besides a few syndromes with identified genetic mutations, the culprit molecular pathway affecting the radiosensitivity of phenotypically normal patients remains elusive (34). For mildly and moderate overreacting patients, polymorphic genetic variations in DNA repair and other processes are believed to be related to the interindividual reactions to radiotherapy (35, 36).

The genomic basis of radiosensitivity is important both in cancer therapy, where normal and tumor cells differ in their response to treatment (37), and in neoplastic transformation, where exposure to radiation (as in occupational and diagnostic radiology) and environmental hazards may have different carcinogenic susceptibilities in the population (38). In fact, DSB repair fidelity is believed to be an important mechanism for radiation-induced cancer and a potential marker for radiation susceptibility. However, both NC and RS are breast cancer patients, and ATM is known to predispose to a certain type of malignancies. Thus, the lowest residual level of DSB misrepaired observed in the NC cells could reflect cancer susceptibility in this breast cancer patient with normal radiosensitivity. Furthermore, the striking observation of the larger amount of misrepaired DSBs in the *Not*I repair fidelity assay compared with the modest amount of unrepaired DSBs in the whole genome (*Alu* assay) suggests that DNA repair fidelity is also a mechanism involved in radiation sensitivity. Therefore, it can be speculated that assessing DNA repair fidelity in cancer patients may be a useful indicator as a prognostic or predictive marker for the likelihood of developing radiotoxicity after radiation treatment or exposure to other clastogenic agents.

## Conclusions

This study demonstrated that in the cells derived from this patient who had severe adverse reactions to radiotherapy, DNA DSBs were more likely to be misrepaired rather than unrepaired. This may imply that DNA repair fidelity is a mechanism leading to adverse reactions to radiotherapy. More studies with large patients' cohort are required to confirm these results. The DNA repair fidelity assay may be an important endpoint to be further perused and developed as a hallmark for radiosensitivity.

## Data Availability Statement

The original contributions generated for this study are included in the article/supplementary material, further inquiries can be directed to the corresponding author/s.

## Ethics Statement

The studies involving human participants were reviewed and approved by Ethics Committee of the Institutional Review Board (CA-06294/16672/50192). The patients/participants provided their written informed consent to participate in this study.

## Author Contributions

MS and GA: conceptualization and funding acquisition. GA and SI: methodology. GA, SI, and NA-H: investigation. GA: formal analysis. MS and GA: validation. GA: writing the original draft. All authors: review and editing.

## Conflict of Interest

The authors declare that the research was conducted in the absence of any commercial or financial relationships that could be construed as a potential conflict of interest.
